# Production of Bioactive Secondary Metabolites by Marine *Vibrionaceae*

**DOI:** 10.3390/md9091440

**Published:** 2011-08-25

**Authors:** Maria Mansson, Lone Gram, Thomas O. Larsen

**Affiliations:** 1 Center from Microbial Biotechnology, Department of Systems Biology, Technical University of Denmark, Søltofts Plads 221, DK-2800 Kgs. Lyngby, Denmark; E-Mail: tol@bio.dtu.dk; 2 National Food Institute, Technical University of Denmark, Søltofts Plads 221, DK-2800 Kgs. Lyngby, Denmark; E-Mail: gram@food.dtu.dk

**Keywords:** *Vibrio*, marine bacteria, bioactive, antibiotics, siderophores

## Abstract

Bacteria belonging to the *Vibrionaceae* family are widespread in the marine environment. Today, 128 species of vibrios are known. Several of them are infamous for their pathogenicity or symbiotic relationships. Despite their ability to interact with eukaryotes, the vibrios are greatly underexplored for their ability to produce bioactive secondary metabolites and studies have been limited to only a few species. Most of the compounds isolated from vibrios so far are non-ribosomal peptides or hybrids thereof, with examples of N-containing compounds produced independent of nonribosomal peptide synthetases (NRPS). Though covering a limited chemical space, vibrios produce compounds with attractive biological activities, including antibacterial, anticancer, and antivirulence activities. This review highlights some of the most interesting structures from this group of bacteria. Many compounds found in vibrios have also been isolated from other distantly related bacteria. This cosmopolitan occurrence of metabolites indicates a high incidence of horizontal gene transfer, which raises interesting questions concerning the ecological function of some of these molecules. This account underlines the pending potential for exploring new bacterial sources of bioactive compounds and the challenges related to their investigation.

## 1. Introduction

**Figure 1:**
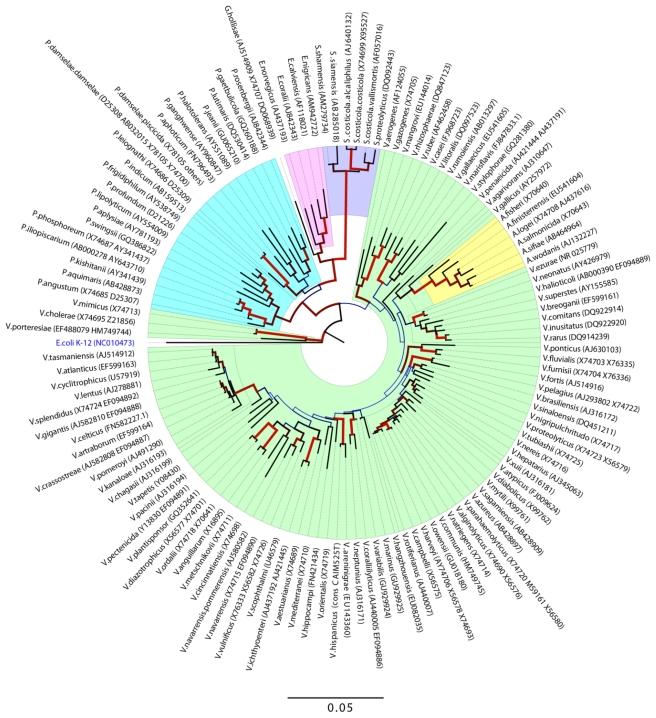
Evolutionary relationship of the *Vibrionaceae* family [3–7].

Marine *Vibrionaceae* are Gram-negative, rod-shaped γ-proteobacteria that are usually motile and possess a chemoheterotrophic metabolism [1]. Members of this family are widespread in the marine environment, including estuaries, coastal waters, and sediments [1]. At this writing, the family includes seven genera (Figure 1): *Allivibrio* (6 species), *Enterovibrio* (4 species), *Salinivibrio* (6 species), *Catenococcus* (1 species), *Grimontia* (1 species), *Vibrio* (89 species), and *Photobacterium* (21 species) [1,2].

### 1.1. Occurrence and Ecological Significance

**Figure 2:**
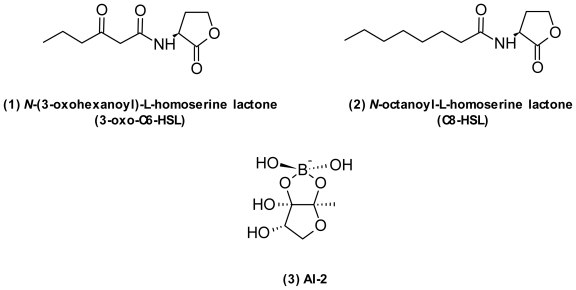
Structures common quorum sensing molecules from *Vibrio* sp.

Vibrios are particularly abundant on the surface of marine macroorganisms such as corals, fish, seagrass, sponges, and zooplankton, where they form commensal, symbiotic, or pathogenic associations [1]. Excellent books and reviews have been published on the taxonomy [8], ecology [1,9,10], and pathogenesis of vibrios [11–14]. Several species are well studied and serve as model systems for understanding symbioses [15,16], interspecies signaling [17], and pathogen persistence [11,18]. A particularly famed vibrio is *Vibrio fischeri*, known for its light organ symbiosis with the Hawaiian squid *Euprymna scolopes* [15]. *V. fischeri* colonizes the squid light organ and provides bioluminescence for the squid to use as countershading in order to evade predators. In return, the bacteria gain a protected nutrient environment. The discovery of *N*-acylhomoserine lactones (Figure 2) as quorum sensing signals was first made in *V. fischeri* and the LuxI/R system of *V. fischeri* is the paradigm of Gram-negative QS systems even though it is not found in all vibrios [18]. In *V. fischeri*, there are three distinct QS signals; 3-oxo-C6-HSL (**1**), C8-HSL (**2**), and AI-2 (**3**). These are used to control a regulatory cascade leading to induction of luminescence. Vibrios also use QS to control traits such as virulence and biofilm formation [17].

The *Vibrionaceae* include species that are opportunistic pathogens of humans and marine animals. *V. vulnificus*, *V. parahaemolyticus*, and *V. cholerae* are serious human pathogens. *V. cholerae* probably has the greatest impact on human health, causing the acute diarrheal disease cholera that can result in epidemics [11]. It is a very persistent bacterium that can survive on a variety of vectors, including zooplankton [19] and cyanobacteria [18]. *V. parahaemolyticus* [20] and *V. vulnificus* [12] are food-borne pathogens associated with the ingestion of raw seafood. *V. anguillarum*, *V. salmonicida*, and *V. vulnificus* are important fish pathogens and are widespread in aquaculture settings, where conditions seem to enhance their virulence [1].

The ability to form biofilms is widespread among vibrios and plays a significant role in the pathogenicity of *V. cholera* [21], *V. parahaemolyticus,* and *V. vulnificus* [22], as well as in the symbiotic colonization by *V. fischeri* [23,24]. Key proteins include pili, lectins, exopolysaccharides, and components involved in the formation of flagella [22]. Though vibrios share a high number of regulatory systems of biofilm formation, there are differences that could reflect different niche specificity or ecological roles [25]. For example, it appears that vibrios produce species-specific exopolysaccharides, the major component of bacterial biofilms, and often have the potential to produce more than one type [22].

High densities of *Vibrio* and *Photobacterium* on the surface of zooplankton [26] have in part been ascribed to the ability of vibrios to utilize chitin, an *N*-acetyl d-glucosamine polymer in zooplankton exoskeletons, as carbon and nitrogen source [27]. The presence of chitinases and chitinase encoding genes has been confirmed for several members of the family [27,28]. Chitin controls several genetic and physiological characteristics of vibrios [19] including antagonistic activity [29]. Also, vibrios are able to degrade other complex carbohydrates such as fucoidan and laminarin found in algal species [29,30]. Thus, this superior nutrient utilization may be one of the reasons for the ubiquitous presence of vibrios in the marine environment [31].

### 1.2. Genomic Diversity and Phylogeny

In contrast to most γ-proteobacteria, vibrios possess two circular chromosomes [32,33]. Essential functions and housekeeping genes are usually located on the large chromosome ChrI, which is rather constant in size (~3 Mb), while the smaller ChrII is flexible in size, ranging between 0.8–2.4 Mb [9]. ChrII contains accessory genes related to transcriptional regulation, for example, pathogenicity and antimicrobial resistance [9,34]. Genes encoding chitin metabolism and quorum sensing are split between the two chromosomes [9]. The ability of vibrios to vary the copy numbers of the two chromosomes is suspected to be involved in the adaptation to varying environmental conditions [34]. Horizontal gene transfer is involved in the genetic flexibility of vibrios, including transduction by phages, plasmid conjugation [35], and so-called “super-integrons” [36,37]. In addition, Meibom *et al.* (2005) [38] showed that vibrios become naturally competent when grown in the presence of chitin, allowing uptake of free DNA from the environment. Chitin-induced competence has been demonstrated in *V. cholera* [38], *V. vulnificus* [39], and *V. fischeri* [40].

**Figure 3:**
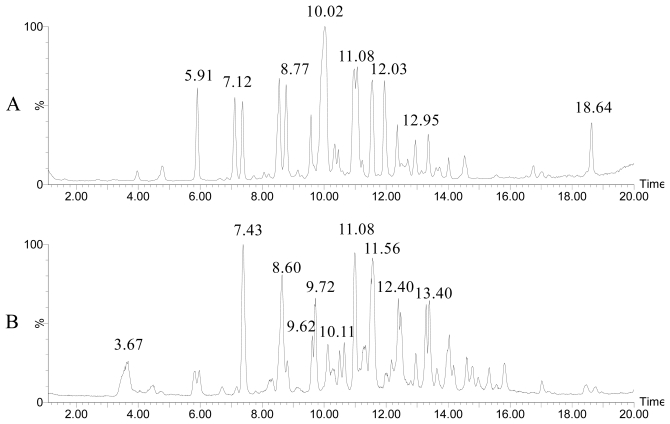
LC-MS profiles of a *V. coralliilyticus* (**A**) and *V. neptunius* (**B**), showing significant differences in secondary metabolite production. Andrimid (RT 10.02) was only found in *V. coralliilyticus* strains. Figure modified from Wietz *et al.* (2010) [45].

The high genomic diversity of vibrios can be directly translated into high phenotypic variability [41]. This makes it difficult to obtain meaningful groupings of vibrios at genus and species level based on isolated phenotypical markers [1]. Also, the 16S rRNA gene is highly conserved, and present in serveral alleles, among the *Vibrionaceae* and not well suited for identification to the species level [41]. Attempts to improve the taxonomy include sequencing and comparison of various housekeeping genes, including *recA*, *rpoA*, *toxR*, which hold greater sequence variability than 16S [41,42]. Taxonomy of vibrios by genetic markers has been supplemented by chemical analyses, including fatty acid methyl ester (FAME) profiling, and more recently by whole-cell MALDI-TOF MS [43,44], and LC-UV/MS chemical profiling [29,45]. Chemotyping was found to be especially useful at sub-species level, identifying differences in antibiotic production [29]. Whole-cell MALDI-TOF MS was able to distinguish closely related species like *V. parahaemolyticus* and *V. alginolyticus* or *V. cholerae* and *V. mimicus* scouting potential biomarkers within a 4000–14,000 Da mass range [43]. Closely related species (*V. coralliilyticus* and *V. neptunius*) could be distinguished based on their secondary metabolite production (Figure 3) [45]. Despite intra-species differences [29], the chemical profiles corroborated the phylogenetic relationship and clearly showed that production of secondary metabolites in vibrios is more than a strain specific trait.

## 2. Natural Product Production by Members of the *Vibrionaceae* Family

Considering their widespread presence in the marine environment and their genomic flexibility, vibrios are largely underexplored for their proclivity to produce secondary metabolites. So far, a total of 93 compounds have been isolated from *Vibrionaceae*. The majority of these compounds have been isolated from only three species; *V. parahaemolyticus*, *V. anguillarum*, and *V. vulnificus*, which is likely a reflection of their importance as pathogens. In the following, all metabolites reported from *Vibrionaceae* (Table 1) will be presented and interesting compounds highlighted in an attempt to give an overview of the chemical diversity and assess the biosynthetic potential of this group of bacteria.

### 2.1. Compounds with Antibacterial Activity

Some marine vibrios produce antibacterial compounds [91–93] that are believed to contribute to their abundance in surface-associated communities [94]. Long and Azam (2001) [92] studied anta-gonistic interactions among pelagic bacteria and found that vibrios produced broad-range antibacterial compounds. Similar capabilities have been observed for coral-associated vibrios [95]. Yet, none of these compounds were isolated and structurally characterized. The relatively widespread production of antibiotics in marine vibrios [45] indicates that antagonistic activity is of ecological importance [29].

**Figure 4:**
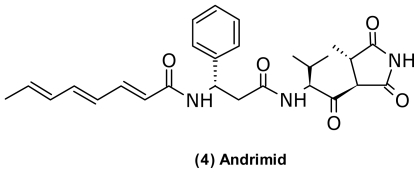
Structure of andrimid isolated from *Vibrio coralliilyticus*.

Probably the best studied antibiotic produced by vibrios is the hybrid NRPS-PKS peptide antibiotic andrimid (**4**) (Figure 4) [46]. The compound interferes with fatty acid biosynthesis [96] and is effective against a wide range of bacteria [97]. Structure-activity studies by Pohlmann *et al.* (2005) [96] revealed that the pyrrolidinedione head was essential for activity, while variations in the fatty acid tail were more tolerable. This suggested that these two structural moieties play different roles in target binding.

Andrimid is a cosmopolitan antibiotic found in distantly related bacteria, including a symbiotic *Enterobacter* sp. from the planthopper *Nilaparvata lugens* [98], *Pseudomonas flourescens* [99], *Pantoea agglomerans* [100], and *Vibrio* sp. [45,46]. The andrimid biosynthetic gene cluster was conserved in two different producers [101]. Interestingly, the cluster encodes resistance genes [102] as well as specific transposases that could be responsible for the diverse occurrence of this antibiotic [100,101]. From *Vibrio* species, the compound was first isolated by Oclarit *et al.* in 1994 [46], and Long *et al.* (2005) [93] identified andrimid as the compound responsible for the growth inhibition of *V. cholerae* by an unidentified *Vibrio* strain. Production of andrimid was for the first time linked to a specific vibrio species by Wietz *et al.* (2010) [45] that isolated the compound from the culture broth of a *V. coralliillyticus* strain S2052. Within *V. coralliilyticus*, the production of andrimid is a marker of different chemotypes [29]. Two *V. coralliilyticus* strains S2052 and S4053 from two distant geographical locations produced andrimid [45], while the type strain and a close relative did not [29]. Interestingly, *V. coralliilyticus* S2052 focused its production of secondary metabolites to the production of andrimid when grown on chitin and also increased the yield of the antibiotic [29]. The bacterium was capable of producing andrimid in a live chitin model system with *Artemia* [29]. This indicated that andrimid potentially contributes to different niche-specificities of *V. coralliilyticus*.

**Figure 5:**
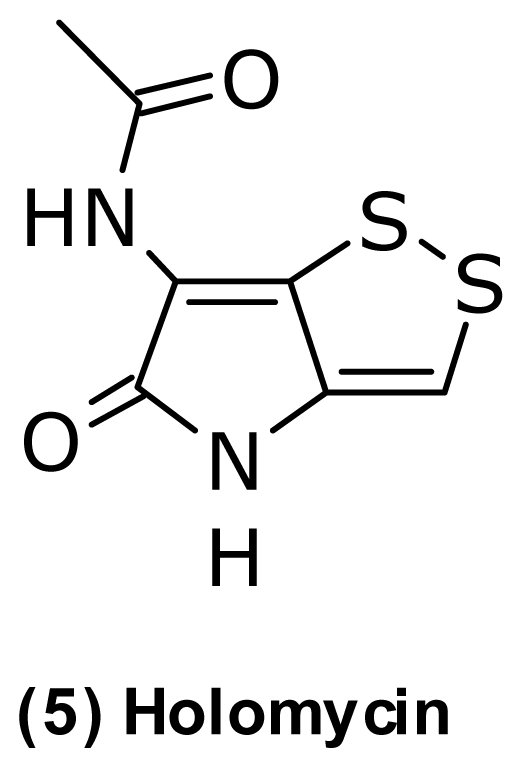
Structure of holomycin isolated from *Photobacterium halotolerans*.

Another example of cosmopolitan antibiotics from *Vibrionaceae* is the highly potent pyrrothine antibiotic, holomycin (**5**) (Figure 5) isolated from a strain closely related to *Photobacterium halotolerans* [45]. Prior to this isolation, holomycin had only been isolated from actinomycetes, including *Streptomyces clavuligerus* [103], *S. griseus* [104], and a marine *Streptomyces* sp. [105].

The NRPS biosynthetic cluster encoding holomycin in *S. clavuligerus* was recently identified by Li and Walsh (2010) [106], and this allows for the comparison of the holomycin clusters in other producers, including *Photobacterium*. Holomycin has a broad spectrum of antibacterial activity against pathogenic bacteria such as *Staphylococcus aureus*, *S. pneumoniae*, *S. epidermis*, *Enterococcus faecalis*, and *Escherichia coli* [107]. The mode-of-action in *E. coli* includes inhibition of RNA chain elongation, but holomycin is suspected to act as prodrug rather than a direct inhibitor of the RNA polymerase [107]. Holomycin is also strongly inhibitory against several marine strains from the *Roseobacter-*clade, *Pseudoalteromonas*, and *Vibrio*, including pathogens such as *V. harveyi*, *V. vulnificus*, and *V. parahaemolyticus* [45], altogether suggesting that holomycin plays a role in antagonism in the marine environment.

**Figure 6:**
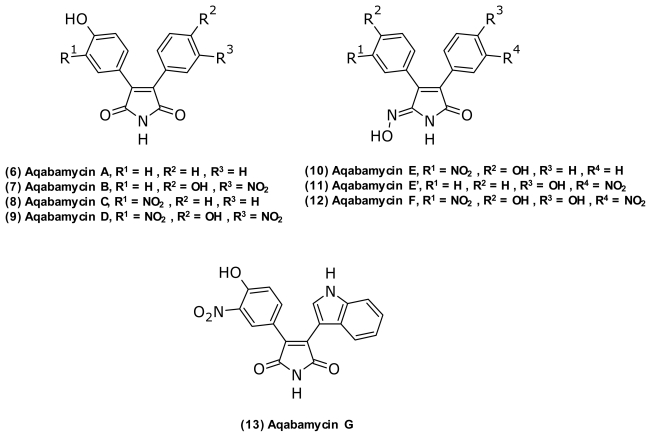
Structures of aqabamycin A–G isolated from coral-associated *Vibrio* sp.

Yao and Al-Zereini recently (2010) [47,48] isolated a series of nitrosubstrituted maleimides called aqabamycins (**6**–**13**) (Figure 6) from a coral-associated *Vibrio* sp. The analogues had varying antibacterial activity against Gram-positive bacteria, including *Micrococcus luteus*, *Bacillus subtilis*, and *B. brevis* as well as cytotoxic activity [48]. The aqabamycins represent a unique structural group both due to their high degree of nitrosubstitution which is rare in nature [108] and the maleimide monoxime present in aqabamycin E/E′ (**10**–**11**) and F (**12**).

**Figure 7:**
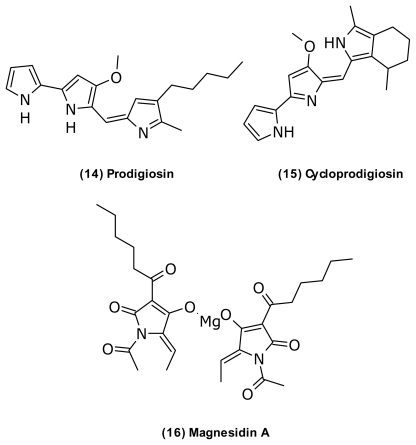
Structures of prodigiosins and magnesidin.

The red pigment and antibiotic prodigiosin (**14**) (Figure 7) has been isolated from *V. psychroerythreus* [60], *V. gazogenes* (originally termed *Beneckea gazogenes* but later revised) [61], and *V. ruber* [62]. Additional producers of this compound include *Alteromonas rubra*/*Pseudoalteromonas rubra* [109,110], *Hahella chejuensis* [111], and different *Serratia* [112], and *Streptomyces* species [113,114]. Prodigiosin and its cyclized analogue (**15**) [50,115] have a broad range of biological activities, including antimicrobial, antimalarial, immunosuppressive, and anticancer [116–118]. Prodiginines have clinical potential in anticancer therapy [118], and prodigiosin is currently in preclinical trials (Aida Pharmaceuticals) for pancreatic cancer [116]. The clinical potential as antibiotics is, however, limited due to a low therapeutic window and considerable toxic effects [119]. Starič *et al.* (2010) [120] recently demonstrated that the production of prodiginines in a *Vibrio* sp. isolated from estuaries conferred competitiveness against a *Bacillus* sp. from the same sample, suggesting that prodigiosin might act as a antibiotic in the natural environment. Interestingly, the prodigiosin producing *V. gazogenes* also produced the unique magnesium containing antibiotic, magnesidin (**16**) (Figure 7) [55,121–123].

**Figure 8:**
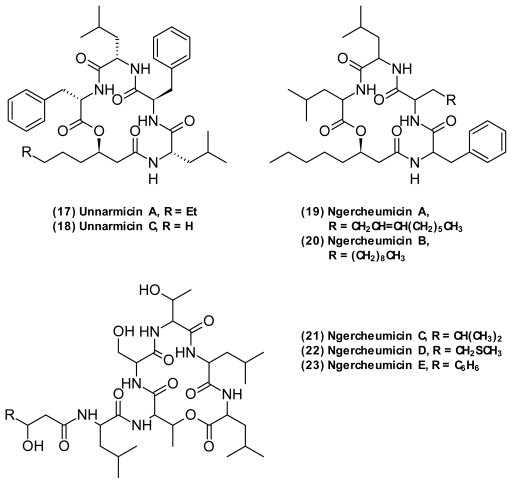
Cyclodepsipeptides isolated from *Photobacterium* sp.

Shizuri and co-workers isolated two distinct groups of depsipeptides (Figure 8), the unnarmicins [63] and ngercheumicins [57] from a *Photobacterium* sp. with potent, but narrow-spectrum antibacterial effect against strains of *Pseudovibrio*. The unnarmicin A (**17**) and C (**18**) consist of four amino acids (l-Phe, l-Leu, d-Phe, l-Leu) and a 3-hydroxyoctanoic and 3-hydroxyhexanoic fatty acid, respectively. The ngercheumicins A–E have a depsipeptide macrocycle and either a fatty acid (**19**–**20**) or peptide tail (**21**–**23**). They have been patented for treating infections caused by *Pseudovibrio denitrificans*, though no literature describes pathogenic traits of this bacterium [124].

### 2.2. Siderophores

Many vibrios produce siderophores as a strategy to sequester iron in the marine environment, where the iron level is extremely low [125,126]. This is necessary to maintain important enzymatic processes (with iron as cofactor) and a prerequisite for pathogenicity for many vibrios. It should be mentioned that siderophores also may have antibacterial activity but are dealt with in a separate section due to their specific metabolic function.

**Figure 9:**
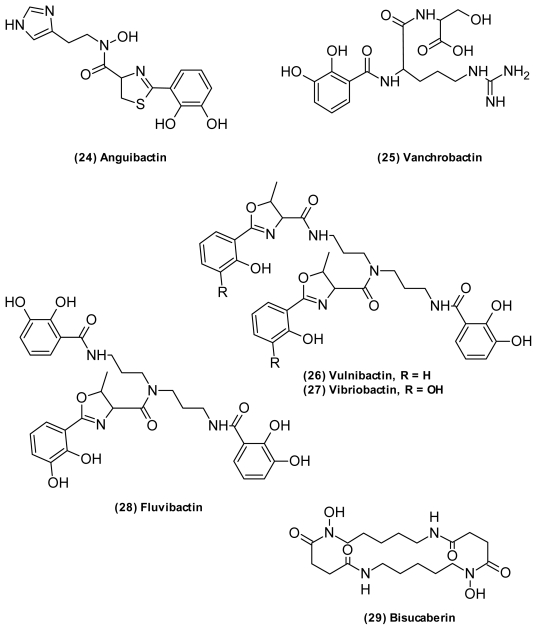
Siderophores isolated from *Vibrio* sp.

A great structural diversity has been observed among the siderophores produced by *Vibrio* species (Figure 9). *V. anguillarum* has at least two different siderophore-mediated systems, namely anguibactin (**24**) [64,127] and vanchrobactin (**25**) [128]. The non-ribosomal peptide anguibactin represents a unique structural class of siderophores with both a catechol and hydroxamate ligand and a thiazole core [64]. The biosynthetic genes encoding this compound are found on a 65-kb virulence plasmid in some *V. anguillarum* strains. Knock-out of genes involved in anguibactin production attenuated virulence, confirming that anguibactin is a prerequisite for successful host-invasion of this bacterium [129]. In contrast, the catechol vanchrobactin is chromosome-encoded, and interestingly, the coding genes are silenced in anguibactin producing strains [71,130]. Recently, dimeric and trimeric versions of vanchrobactin were isolated from an unidentified *Vibrio* by Sandy *et al.* (2010) [65]. Also, they found anguibactin to possess cytotoxic activities against P388 murine leukemia cells [65].

Vibriobactin (**27**) [72], vulnibactin (**26**) [74], and fluvibactin (**28**) (Figure 9) [68] are unique siderophores produced by *V. cholerae*, *V. vulnificus*, and *V. fluvialis*, respectively. They are all catechol hydroxyphenyloxalone siderophores that share a rare norspermidine backbone, giving them a propeller-like structure. In vibriobactin and vulnibactin, two of the hydroxybenzoyl moieties are linked to the backbone through an l-threonine, forming an oxazoline ring. Fluvibactin only has one oxazoline ring, with one hydroxybenzoyl directly linked to the norspermidine terminal. Vibriobactin and vulnibactin differ only in the number of hydroxylations, and this high structural similarity enables cross-utilization of these two siderophores [74].

Bisucaberin (**29**) (Figure 9) [69] is a symmetric cyclic dihydroxamate produced by the fish pathogen *V. salmonicida* [70]. Unlike most other vibrio siderophores [129], bisucaberin is produced through an NRPS-independent route [131] where alternating dicarboxylic acids and diamine or amino alcohols are assembled through amide or ester bonds [132]. Bisucaberin was found to be useful in combinatorial anticancer therapy by sensitizing tumor cells to macrophage-mediated cytolysis [69,133].

Siderophores are used as therapeutic deferration agents to treat iron overload in chronically transfused thalassemia patients. A stereochemically modified version of fluvibactin efficiently removed iron without increasing microbial growth [134]. It has been suggested that siderophores can be used for the development of a new class of “trojan horse” antibiotics [135]. Siderophore-antibiotic conjugates exploit the iron transport system of the pathogen to penetrate the bacterial outer membrane, increasing the antibacterial activity of the antibiotic [136]. Recently, Bergeron *et al.* (2009) [137] made a conjugate linking antibodies to vulnibactin as a strategy towards a vaccine against *V. vulnificus*.

### 2.3. Compounds with Other Activities

**Figure 10:**
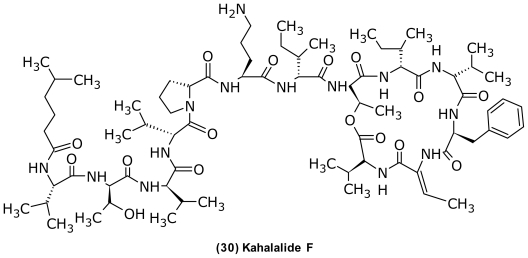
Structure of kahalalide F isolated from *Vibrio mediterranei/shilonii*.

Another interesting group of compounds produced by a member of the *Vibrionaceae* is the kahalalides. These cyclic depsipeptides were originally isolated from the herbivorous mollusc *Elysia refescens* and its diet, the green algae *Bryopsis* sp. In particular, kahalalide F (**30**) (Figure 10) has an attractive spectrum of activities against AIDS-related opportunistic infections and against cancer cell lines [138]. Kahalalide F is currently undergoing Phase II clinical trials (PharmaMar) for the treatment of prostate, lung, and liver cancer [138] and in patients with severe psoriasis (PharmaMar/Marinomed) [139]. Interestingly, Hill and Hamann (2005) [75] found kahalalide F as well as two analogues to be produced by a *V. mediterranei/shilonii*. The finding of a microbial origin for this compound allows for the large-scale industrial fermentation of this compound rather than arduous organic synthesis.

**Figure 11:**
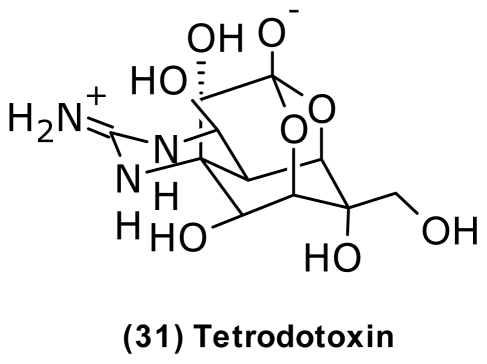
Structure of tetrodotoxin isolated from *Vibrio harveyi* and *Vibrio alginolyticus*.

Several vibrios produce the potent neurotoxin tetrodotoxin (TTX) (**31**) (Figure 11), also known as the pufferfish poison [85]. The true origin of TTX has been the subject of much debate [140], nonetheless *V. harveyi* and *V. alginolyticus* isolated from different species of pufferfish produced the toxin as well as several analogues [84]. Also, *V. fischeri* isolated from the intestines of the xanthid crab, *Atergatis floridus* produced TTX [87]. Vibrios dominated the intestinal microbiota of the pufferfish, *Fugu vermicularis vermicularis* [84], suggesting that the toxification is caused by TTX-producing bacteria accumulated through the food web [85]. The role of these compounds to vibrio itself is still unclear, though it has been suggested to play a role in regulating sodium transport [85].

**Figure 12:**
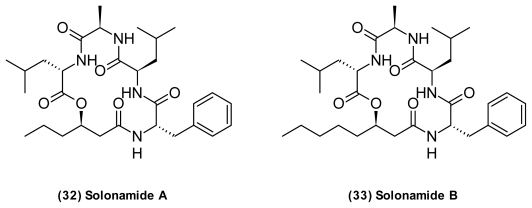
Structures of solonamides isolated from *Photobacterium halotolerans* related strain.

Vibrios produce compounds that interfere with the quorum sensing system of Gram-positive bacteria. From a strain related to *P. halotolerans* two novel depsipeptides (Figure 12), solonamides A and B (**32**–**33**) that interfere with QS regulated virulence genes in *S. aureus* were isolated [83]. In particular, solonamide B dramatically reduced expression of both *hla* encoding α-hemolysin and *RNAIII*, while increasing expression of *spa* encoding Protein A. This suggested that the depsipeptides interfere with *agr*, the global virulence regulator in *S. aureus*. High structural similarity of the solonamides to the natural autoinducers of the *agr* system suggested that they might be competitive inhibitors. Interestingly, the solonamides had a pronounced effect on virulence gene expression in *S. aureus* strain USA300, which is the predominant community-acquired MRSA (CA-MRSA) strain in the USA [141]. The solonamides strongly resemble the unnarmicins (**17**–**18**) found in an unidentified *Photobacterium* sp. (Section 2.3.1) [63]. Thus, it is possible that the unnarmicins also possess QSI activity.

**Figure 13:**
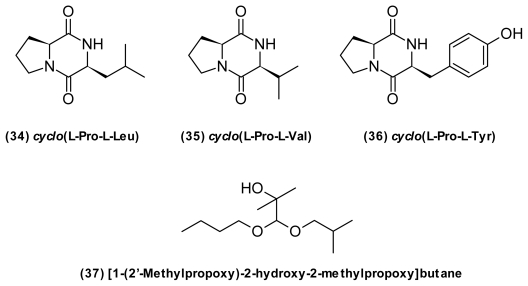
Structures of common diketopiperazines from *Vibrio* sp. and [1-(2′-methylpropoxy)-2-hydroxy-2-methylpropoxy]-butane.

Several small molecules isolated from vibrios induce Gram-negative quorum sensing systems. That includes various diketopiperazines (DKP) (Figure 13). For example, *cyclo*(l-Pro, l-Leu) (**34**), *cyclo*(l-Pro, l-Val) (**35**), and *cyclo*(l-Pro, l-Tyr) (**36**), DKPs commonly isolated from vibrios [53], modulated LuxR-type protein activity though at higher concentrations than AHLs [142]. It is speculated that these dipeptides represent a new class of naturally occurring QS signals potentially involved in interspecies signaling, as DKPs are found in most culturable marine bacteria [143]. However, some DKPs are likely to be artifacts generated from media components during work-up procedures [144]. De Nys *et al.* (2001) [80] isolated [1-(2′-methylpropoxy)-2-hydroxy-2-methylpropoxy]-butane (**37**) (Figure 13) from *P. angustum* (*V. angustum*) S14 with the ability to mediate expression in two AHL-regulated systems, inducing bioluminescence in *V. harveyi* and the AHL reporter system in *Agrobacterium tumefaciens*.

### 2.4. Compounds with Unknown Activities

**Figure 14:**
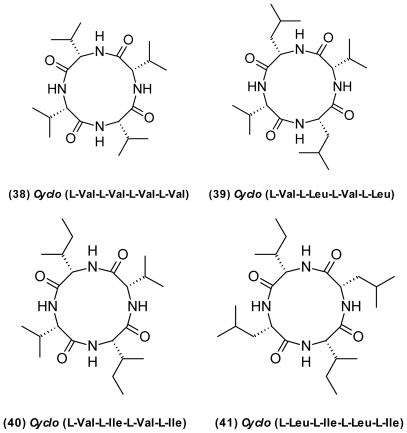
Structures of cyclotetrapeptides isolated from *Photobacterium*.

Vibrios also produce numerous compounds for which no biological activity has been reported so far. That includes small-molecule by-products, for example some nitro-substituted compounds such as 3-nitroindazole and 3-nitro-4-hydroxycinnamic acid [47]. From *P. halotolerans* S2753, we isolated a series of cyclic tetrapeptides (Figure 14); *cyclo*(l-Val-l-Val-l-Val-l-Val) (**38**), *cyclo*(l-Val-l-Leu-lVal- l-Leu) (**39**), *cyclo*(l-Val-l-Ile-l-Val-l-Ile) (**40**), and *cyclo*(l-Leu-l-Ile-l-Leu-l-Ile) (**41**) (Kjaerullf and Mansson, unpublished data). These types of peptides are often found in marine culturable bacteria [145–147], suggesting that they are storage compounds accumulated during growth under excess nutrients.

**Figure 15:**
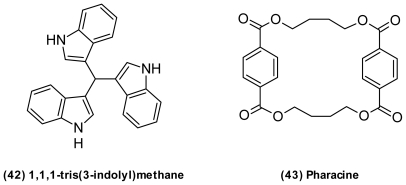
Structures of 1,1,1-tris (3-indolyl) methane and pharacine, examples of potential artefacts from work-up of *Vibrio* extracts.

Many of compounds isolated from vibrios are suspected to be artifacts generated from media components during work-up procedures [54,144,148]. These include several bis- and trisindole derivatives from a *V. parahaemolyticus* strain, Bio249 [54]. An example is 1,1,1-tris (3-indolyl) methane (**42**) (Figure 15) that could easily be formed by simple condensation of indole-3-carbaldehyde and indole, both having been isolated from *V. parahaemolyticus* [54]. From the same *V. parahaemolyticus* strain, the cyclic terephthalic acid ester, pharacine (**43**) [148] was isolated [54]. This was suspected to be an artifact from plastic material contaminants; however, fermentation results were reproducible with no contact with plastic. Until biosynthetic studies have been performed, the true origin of these molecules remains uncertain.

## 3. Conclusion

The versatility and widespread occurrence of vibrios can be ascribed to different characteristics such as their superior nutrient utilization, their excellent biofilm formation, and their genetic construction. High genomic flexibility in *Vibrionaceae* makes this group of bacteria very apt to resist various environmental changes, for example through the acquisition of biosynthetic genes linked to the production of antibiotics or siderophores. So, rampant horizontal gene transfer occurs in these bacteria. As a reflection, most compounds isolated from vibrios have also been found in other types of bacteria, in many cases from distantly related taxa [45]. Sometimes the mobile genetic elements are even incorporated in the biosynthetic cluster itself, making it even more prone to gene-exchange. The antibiotic andrimid is an example of a compound encoded by such a “nomadic gene cluster” [149].

Production of secondary metabolites in vibrios has been linked to antagonism, intraspecies communication, and pathogenicity. The compounds produced by vibrios are mainly non-ribosomal peptides or hybrids hereof, with examples of *N*-containing compounds produced by NRPS-independent pathways. Despite this narrow structural span compared to metabolites produced by other marine bacteria, vibrios produce compounds with a broad range of interesting biological activities. For example the solonamides, cyclic depsipeptides from *P. halotolerans* were found to attenuate virulence in a CA-MRSA strain [83] and the cyclic depsipeptide kahalalide F from *V. medierranei* [75] that is undergoing Phase II clinical trials for the treatment of prostate, lung, and liver cancer [138].

Many vibrios have multiple lifestyles, including a planktonic (free swimming), sessile (attached to zooplankton or other surfaces), and a pathogenic form [1]. As production of secondary metabolites often confers a selective advantage to the producing organism [150], the diverse lifestyles of these bacteria are reflected in their metabolic capabilities. There are intraspecies variations in the compounds produced, with different chemotypes potentially reflecting niche adaptation. For example, antagonistic strains of *V. coralliilyticus* were found to produce andrimid in high yields, while pathogenic related strains did not have the ability to produce the antibiotic [29].

The cosmopolitan occurrence of several vibrio metabolites raises the question whether there are unique *Vibrionaceae* metabolites. Of the 227 vibrio genomes sequenced so far [2,151], only a fraction has been fully assembled [33], mainly pathogenic *V. cholerae* strains [9], and none have been functionally annotated with regard to the presence of biosynthetic clusters. Thus, it is still uncertain whether these bacteria represent a novel “hotspot” of secondary metabolites. For the future, it will be of utmost interest to extend full-genome sequencing to other vibrios and investigate the prevalence of biosynthetic genes linked to secondary metabolism. Also, this will make it possible to compare homology of biosynthetic genes between diverse producers of cosmopolitan antibiotics. Overall, this will allow insight into the ecological roles of these bacteria and the environmental and physiological parameters governing production of their secondary metabolites.

## Figures and Tables

**Table 1:** Bioactive compounds produced by marine *Vibrionaceae*. Excluded from the list are sugars, fatty acids, and small peptides commonly found in marine culturable bacteria. Excluded are also compounds from AntiBase 2010 whose presence could not be confirmed in any reference referring to *Vibrionaceae*.

Bioactivities	Name	Compound class	Source	Other activities	Ref.
Antibacterial	Andrimid (**4**)	Pyrrolidinedione	*V. coralliilyticus*		[45,46]
	Aqabamycin A (**6**)	Nitro maleimide	*Vibrio* sp.	Anticancer	[47,48]
	Aqabamycin B (**7**)				
	Aqabamycin C (**8**)				
	Aqabamycin D (**9**)				
	Aqabamycin E (**10**)	Maleimide oxime			
	Aqabamycin E’ (**11**)				
	Aqabamycin F (**12**)				
	Aqabamycin G (**13**)	Nitro maleimide			
	B-4607-C	Phenazine	*Vibrio* sp.		[49]
	Cycloprodigiosin (**15**)	Prodiginine	*V. gazogenes*		[50]
	3,5-Dibromo-2-(3′,5′-dibromo-2′-methoxyphenoxy)-phenol	Diphenyl ether	*Vibrio* sp.	Antifungal	[51,52]
	2,2-Di-(3-indolyl)-3-indolone	Indole	*V. parahaemolyticus*		[53,54]
	Griseoluteic acid	Phenazine	*Vibrio* sp.		[49]
	Holomycin (**5**)	Pyrrothine	*P. halotolerans*		[45]
	Indazole-3-carbaldehyde	Indazole	*Vibrio* sp.	Anticancer	[47]
	Magnesidin A (**16**)	Tetramic acid Mg^2+^ salt	*V. gazogenes*	Antialgal	[55]
	Moiramide B	Pyrrolidinedione	*Vibrio* sp.		[56]
	Ngercheumicin A (**19**)	Depsipeptide	*Photobacterium* sp.		[57]
	Ngercheumicin B (**20**)				
	Ngercheumicin C (**21**)				
	Ngercheumicin D (**22**)				
	Ngercheumicin E (**23**)				
	Pelagiomicin C	Phenazine	*Vibrio* sp.	Anticancer	[49,58,59]
	Prodigiosin (**14**)	Prodiginine	*V. psychroerythrus* *V. gazogenes* *V. ruber*	Antiprotozoan antifungal anticancer	[60–62]
	Turbomycin	Indole	*Vibrio* sp. (*V. parahaemolyticus*)	Antifungal	[54]
	Unnarmicin A (**17**)	Depsipeptide	*Photobacterium* sp.	Antifungal	[63]
	Unnarmicin C (**18**)				
	Vibrindole A	Indole	*V. parahaemolyticus*	Antifungal	[53]
Siderophore	Anguibactin (**24**)	Catechol hydroxamate	*V. anguillarum*	Anticancer	[64,65]
	Aerobactin	Hydroxamate	*Vibrio* sp.		[66]
	Amphibactin B	Hydroxamate (amphiphilic)	*Vibrio* sp.		[67]
	Amphibactin C				
	Amphibactin D				
	Amphibactin E				
	Amphibactin F				
	Amphibactin G				
	Amphibactin H				
	Amphibactin I				
	Bis-[3-(2,3-dihydroxybenzoylamino)-propyl]-amin	Catechol	*V. fluvialis*		[68]
	Bisucaberin (**29**)	Hydroxamate	*V. salmonicida*	Anticancer	[69,70]
	Divanchrobactin	Catechol	*Vibrio* sp.		[65]
	Fluvibactin (**28**)	Catechol Hydroxyphenyloxazolone	*V. fluvialis*		[66]
	Trivanchrobactin	Catechol	*Vibrio* sp.		[65]
	Vanchrobactin (**25**)	Catechol	*V. anguillarum*		[71]
	Vibriobactin (**27**)	Catechol Hydroxyphenyloxazolone	*V. cholerae*		[72]
	Vibrioferrin	Carboxylate	*V. parahaemolyticus*		[73]
	Vulnibactin (**26**)	Catechol Hydroxyphenyloxazolone	*V. vulnificus*		[74]
	Vulnibactin 2	Vulnibactin precursor			
	Vulnibactin 3				
Anticancer	Kahalalide F (**30**)	Depsipeptide	*V. mediterranei* (*V. shilonii*)	Antibacterial antimalarial antifungal	[75]
	Kahalalide H				[76]
	Kahalalide J				
Quorum sensing interference	AI-2 (**3**)	Furanosyl borate diester	*Vibrio*	QS	[76]
	*N*-hexanoyl-l-homoserine lactone	Homoserine lactone	*V. anguillarum*	QS	[77]
	*N*-(3-hydroxybutanoyl)-l-homoserine lactone	Homoserine lactone	*V. harveyi*	QS	[78]
	*N*-(3-hydroxyhexanoyl)-l-homoserine lactone	Homoserine lactone	*V. anguillarum*	QS	[79]
	[1-(2′-methylpropoxy)-2-hydroxy-2-methylpropoxy] butane (**41**)		*P. angustum* (*V. angustum*)	QS	[80]
	*N*-(3-oxodecanoyl)-l-homoserine lactone	Homoserine lactone	*V. anguillarum*	QS	[81]
	*N*-(3-oxohexanoyl)-l-homoserine lactone (**1**)	Homoserine lactone	*V. fischeri* *V. cholerae* *V. harveyi* *V. anguillarum*	QS	[17,82]
	*N*-octanoyl-l-homoserine lactone (**2**)	Homoserine lactone	*V. fischeri*	QS	[77]
	Solonamide A (**32**)	Depsipeptide	*P. halotolerans*	QSI Gram pos	[83]
	Solonamide B (**33**)				
Na channel blocker	Anhydro-tetrodotoxin		*Vibrio* sp.		[84,85]
	4-epi-tetrodotoxin		*Vibrio* sp.		[84,85]
	Tetrodonic acid		*Vibrio* sp.		[85,86]
	Tetrodotoxin (**31**)		*V. harveyi* *V. alginolyticus* *V. fischeri*		[84,85,87]
Riboflavin synthase inhibitor	7-hydroxy-6-methyl-8-(1-d-ribityl)lumazine	Pteridine	*P. phosphoreum*		[88]
	Photolumazine A				
	Photolumazine B				
	Photolumazine C				
Misc.	Arundine	Indole	*V. parahaemolyticus*		[54]
	Benzoic acid	Aromatic	*Vibrio* sp.		[47]
	3,3-Bis-(3-indolyl)butan-2-one	Indole	*V. parahaemolyticus*		[54]
	3,3′-Bisindolylmethane				
	1,4-dithiane		*Vibrio* sp.		[47]
	3-hydroxybenzoic acid	Aromatic	*Vibrio* sp.		[47]
	4-hydroxycinnamic acid				
	p-Hydroxyphenyl-acetamide	Aromatic	*V. parahaemolyticus*		[54]
	Indole-3-carboxaldehyde	Indole	*V. parahaemolyticus*		[53]
	Indole-3-acetic acid	Indole	*Vibrio* sp.		[89]
	6-methyl-8-d-ribityl-2,4,7-trioxopteridine	Pteridine	*P. phosphoreum*		[90]
	3-nitro-4-hydroxy-benzaldehyde	Nitro aromatic	*Vibrio* sp.		[47]
	3-nitro-4-hydroxycinnamic acid				
	3-nitro-1*H*-indazole				
	Pharacine (**43**)	Terephthalic ester	*V. parahaemolyticus*		[54]
	Phenylacetic acid	Aromatic	*Vibrio* sp.		[47]
	Phenyl-2-bis-indolylmethane	Indol			
	Photopterin A	Pteridine	*P. phosphoreum*		[90]
	8-d-ribityl-2,4,7-trioxopteridine				
	Trisindoline	Indole	*V. parahaemolyticus*		[54]
	1,1,3-Tris-(3-indolyl)butane				
	1,1′,1″-Trisindolyl-methane (**42**)				
